# Neuropsychiatric Systemic Lupus Erythematosus With Early-Onset Brain Atrophy: A Case Report and Literature Review

**DOI:** 10.7759/cureus.93751

**Published:** 2025-10-03

**Authors:** Khaled S AlDairi, Raja A Bakhsh, Jouvany S Naguib, Sarab S Alharthi, Manayer G Almutairi, Lama R Alzubaidi, Rayan K Elamin Hassabelrasoul, Ossama S Alhindi, Raheel A Alharazi

**Affiliations:** 1 Department of Internal Medicine, King Faisal Hospital, Makkah, SAU; 2 Department of Internal Medicine, Assiut University Hospitals, Assiut, EGY; 3 Department of Internal Medicine, King Faisal Hospital, Jeddah, SAU; 4 Medicine, King Abdelaziz University Hospital, Jeddah, SAU; 5 Department of Internal Medicine, Al-Sulayyil General Hospital, As Sulayyil, SAU

**Keywords:** brain atrophy, generalized tonic clonic seizures, immunosuppressive treatment, neuropsychiatric systemic lupus erythematosus (npsle), systemic lupus erythematosus

## Abstract

Neuropsychiatric systemic lupus erythematosus (NPSLE) is a serious condition with varied manifestations of systemic lupus erythematosus (SLE) that involves the central and peripheral nervous systems. While seizures and psychiatric components are common findings, brain atrophy is not frequently reported, especially in young individuals. We present a case of a 22-year-old Saudi female who presented with a generalized tonic-clonic seizure and a significant history of undiagnosed psychiatric suffering. Neuroimaging of the brain demonstrated cortical atrophy and extensive subcortical hyperintensities. Autoimmune serology revealed SLE with the positivity of anti-dsDNA and anticardiolipin antibodies. The patient was diagnosed with NPSLE and treated with corticosteroids, hydroxychloroquine, and rituximab. A one-year follow-up MRI showed radiological stability with no new lesions or progression of atrophy. Clinically, she achieved complete seizure remission and improved neurocognitive function.

We performed a structured literature review following PRISMA (Preferred Reporting Items for Systematic reviews and Meta-Analyses) guidelines. Databases were searched, including PubMed, Google Scholar, Springer, and Web of Science, for publications between 2013 and 2025. A total of 1,770 results were screened, and 20 studies were included in the review. The literature review and the case that is discussed support the link between neuroinflammation, injury mediated by autoantibodies, and structural brain changes in NPSLE. Seizures are regularly observed; cortical atrophy is atypical but could represent significant, long-term damage, especially among adults. Immunosuppressive therapy with rituximab can help stabilize both radiological findings and clinically significant manifestations of the disease.

This report highlights the necessity of early recognition of neuropsychiatric symptoms in young individuals with an undiagnosed SLE. If an MRI shows central nervous system (CNS) atrophy, then aggressive immunosuppressive therapy is warranted despite the rare nature of findings during clinical presentation. Longitudinal imaging combined with multidisciplinary care may be particularly appropriate, as coordination among the neurologist, rheumatologist, and primary care physician is essential to achieving favorable outcomes in NPSLE.

## Introduction

Systemic lupus erythematosus (SLE) is a chronic autoimmune disease with multi-organ involvement and a vast array of clinical presentations. Neuropsychiatric systemic lupus erythematosus (NPSLE) is probably one of the most severe variants, encompassing a broad range of neurological and psychiatric syndromes affecting the central and peripheral nervous systems [1]. NPSLE presents as an array of symptoms, such as seizures, cognitive dysfunction, psychosis, mood disorder, or, less frequently, as structural changes to the brain such as cerebral or hippocampal atrophy [2]. Seizures appear in 10-20% of patients with SLE, and sometimes they are the initial manifestation of neuroinflammatory involvement [3].

Brain atrophy, mostly linked to aging and neurodegenerative diseases, has also been described in young SLE patients who more often have neurocognitive decline or neuroinflammation of long duration [4-6]. The proposed mechanisms of neuropsychiatric involvement in SLE include autoantibodies and proinflammatory cytokines (for instance, IL-6 and TNF-α), which impair the blood-brain barrier (BBB), allowing central nervous system (CNS) immune factors to enter the CNS. Consequently, neuroinflammation occurs, which includes microglial activation and neuronal injury, reflected clinically as a variety of neuropsychiatric disorders, which include seizures, cognitive dysfunction, and mood disorders.

In the neuroimaging studies, particularly MRI, a subtle or progressive change in cerebral morphology is detected. Several researchers have reported regional and global decreases in brain volume in SLE patients, even in the absence of overt neuropsychiatric symptoms [7,8]. When severe, NPSLE can show up on MRI as diffuse cortical atrophy or periventricular white matter hyperintensities, hippocampal lesions, or might even include ventriculitis [9,10]. We discuss the case of a 22-year-old Saudi female who presented with status epilepticus and cognitive changes and was later diagnosed with NPSLE. MRI showed diffuse cortical atrophy and white matter changes, and follow-up imaging one year later revealed disease stabilization after immunosuppressive therapy. This report contributes to the current literature regarding the prevalence, pathophysiology, imaging findings, and treatment outcomes of patients with NPSLE and cerebral atrophy.

## Case presentation

History and complaint

A 22-year-old Saudi female, with no known past medical history, was brought to the emergency department by her family following a generalized tonic-clonic seizure. She had received all her childhood vaccines, and there was no family history of autoimmune disease. She had been born without any complications. She was a non-smoker, did not take any regular medications, was single, had no history of recent travel, kept no pets at home, and had no history of blood transfusions or previous surgeries.

The episode had begun while she was cooking, with a sudden collapse, full-body jerking movements, tongue biting, and hypersalivation. There was no reported history of recent fever, trauma, stress, sleep deprivation, or prior seizures. Her early history was unremarkable except for complaints of recurrent oral ulcers, episodic fatigue, and nonspecific arthralgia over the previous years.

Notably, since the age of 13 years, the patient had exhibited signs of psychiatric disturbance, including auditory and visual hallucinations, emotional lability, withdrawal from social interaction, and declining cognitive abilities. These had not been previously evaluated. Family members reported a progressive change in the patient’s baseline mental and cognitive function over the years, culminating in poor general knowledge and impaired calculation abilities.

Examination

Initial evaluation in the emergency department showed stable vital signs with a blood pressure of 120/75 mmHg, a heart rate of 86 bpm, a respiratory rate of 18 breaths per minute (assisted ventilation), an oxygen saturation of 97% on mechanical ventilation, and a temperature of 36.8 °C. She was sedated and intubated, thus limiting detailed neurological assessment. However, her tone and reflexes were normal, and there was no neck rigidity or meningeal irritation. At the emergency department, the patient was seen after sedation and intubation. The neurological assessment was not done properly, as the patient was on sedation.

However, there was no asymmetrical face or a focal neurological deficit. The examination revealed normal tone in the upper and lower limbs and no neck rigidity. The chest was clear bilaterally, and the cardiac exam showed a regular heartbeat. Also, the abdomen was soft and lax with no organomegaly. Lower limb examination showed no edema, skin rashes, or signs of deep vein thrombosis (DVT). The patient was admitted to the ICU for three days as a case of status epilepticus and possible meningoencephalitis. The family refused permission for a lumbar puncture; the patient was given the antiepileptic levetiracetam 750 mg IV bid and started empirically on antibiotics.

Investigations

Baseline laboratory investigations were conducted on admission, and the results are summarized in Table 1, along with corresponding reference ranges and clinical interpretations to facilitate assessment of abnormalities relevant to SLE.

**Table 1:** Baseline laboratory investigations on admission with corresponding reference ranges and clinical interpretations Laboratory workup revealed lymphocytic leukocytosis and elevated ESR and CRP. Infectious and metabolic screens, including blood, urine, and sputum cultures, as well as serologic testing for brucellosis, syphilis, and HIV, were negative. Autoimmune screening showed a positive ANA (1:80, homogenous pattern), anti-dsDNA, and anticardiolipin antibodies. Complement levels (C3, C4) were normal WBC: white blood cells; AST: aspartate aminotransferase; ESR: erythrocyte sedimentation rate; CRP: C-reactive protein; VDRL: Venereal Disease Research Laboratory; HIV: human immunodeficiency virus; ANA: antinuclear antibody; SLE: systemic lupus erythematosus

Test	Result	Normal range	Interpretation
WBC	12.3 ×10⁹/L (mainly lymphocytic)	4.0–11.0 ×10⁹/L	Mild leukocytosis
Hemoglobin (HGB)	11.2 g/dL	12–16 g/dL (female)	Mild anemia
Platelets (PLT)	461 ×10⁹/L	150–450 ×10⁹/L	Borderline thrombocytosis
Renal panel	Within normal limits	—	Normal kidney function
Liver panel – AST	50 U/L	10–40 U/L	Mild elevation
Liver panel – other values	Within normal limits	—	Normal
Cardiac panel – CKI	267 U/L	25–200 U/L	Elevated
ESR	75 mm/hr	<20 mm/hr	Markedly elevated
CRP	5.9 mg/L	<5 mg/L	Slightly elevated
Immunoglobulins (IgA, IgG, IgM)	Normal levels	—	Normal
C3 and C4	Normal	—	Normal complement levels
Direct antiglobulin test (DAT)	Negative	Negative	Normal
Blood culture	Negative	Negative	No growth
Urine culture	Negative	Negative	No growth
Sputum culture	Negative	Negative	No growth
Brucellosis titer	Negative	Negative	Normal
VDRL (syphilis)	Negative	Negative	Non-reactive
HIV	Negative	Negative	Negative
Urine protein/creatinine ratio	53.8 mg/mmol (5.4 g/day of proteinuria)	<15 mg/mmol	Significant proteinuria
ANA	Positive (1:80, homogeneous pattern)	Negative	Autoimmune activity
Anti-dsDNA	Positive	Negative	Specific for SLE
Antiphospholipid antibodies	Positive	Negative	Suggestive of antiphospholipid syndrome

A post-extubation mental-status examination revealed a woman appearing younger than her stated age with below-average grooming. She was calm and cooperative with fair eye contact. No hallucinations or other perceptual disturbances were observed. Comprehension was reduced; she often required repetition of questions and, at times, provided brief or indirect answers. Speech was generally coherent and goal-directed, with intermittent tangentiality. Affect was reactive, occasionally incongruent with content. She was conscious and alert; attention was adequate; and orientation to time, place, and person was intact. Cognitive screening showed impaired calculation and a limited fund of knowledge. As for seizure course, no events occurred during the first 72 hours, followed by a single one-minute event on hospital day four. Despite broad-spectrum antibiotics for presumed meningitis, inflammatory markers remained elevated, and cultures were negative, increasing suspicion for an autoimmune etiology of her neurological presentation.

An initial brain CT revealed diffuse cortical atrophy without signs of infarction or hemorrhage. An MRI of the brain (May 2024) showed extensive periventricular and deep white matter FLAIR hyperintensities, bilateral thalamic and pontine signal abnormalities, and diffuse cortical atrophy with an enlarged ventricular system disproportionate to her age. These findings raised suspicion for inflammatory or autoimmune etiology, including demyelinating disease or vasculitis.

**Figure 1:**
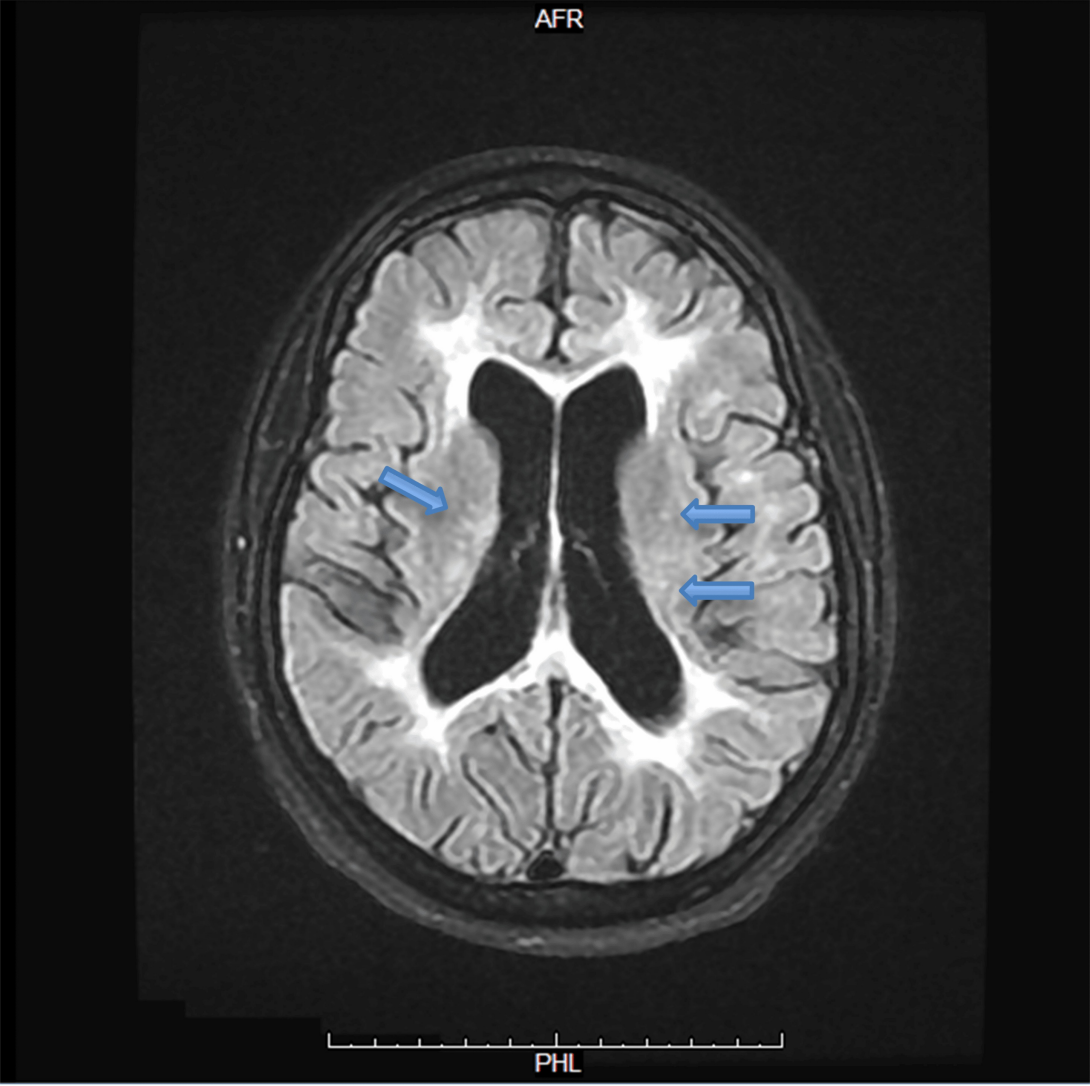
Axial T2 FLAIR MRI of the brain without contrast Axial T2 FLAIR brain MRI shows hyperintense white matter lesions seen around the periventricular regions bilaterally (blue arrows), extending along the lateral ventricles. The distribution appears symmetrical. The ventricles themselves look mildly prominent, but there is no evidence of acute hemorrhage, mass effect, or midline shift. The cortical sulci and gray–white matter differentiation are preserved MRI: magnetic resonance imaging; FLAIR: fluid attenuated inversion recovery

**Figure 2:**
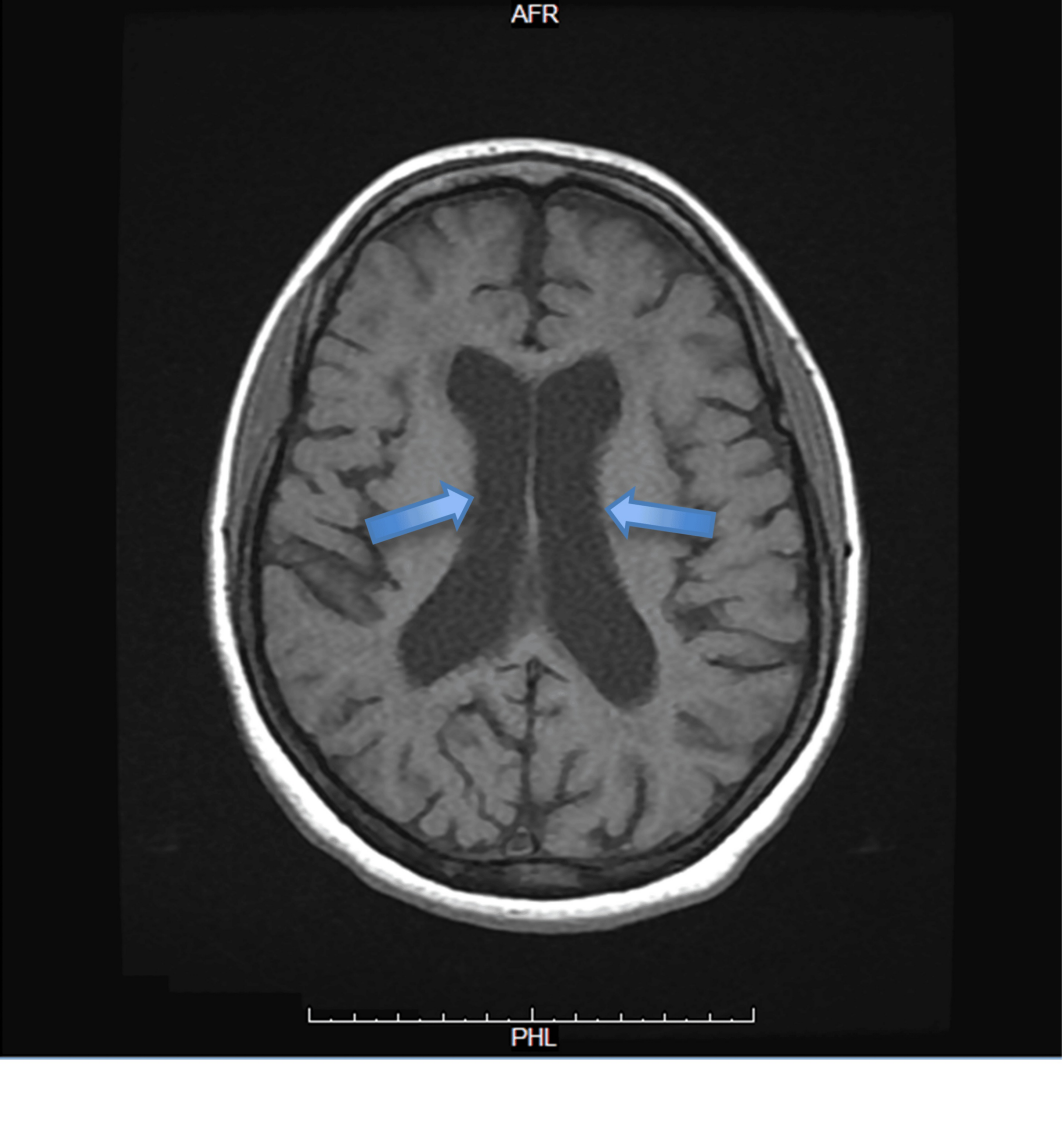
Axial T1-weighted MRI of the brain without contrast This axial T1-weighted MRI demonstrates prominent lateral ventricles (blue arrows) and diffuse cortical sulcal widening, indicating cortical atrophy disproportionate to patient age. The periventricular and deep white matter appear hypointense (darker on T1) MRI: magnetic resonance imaging

**Figure 3:**
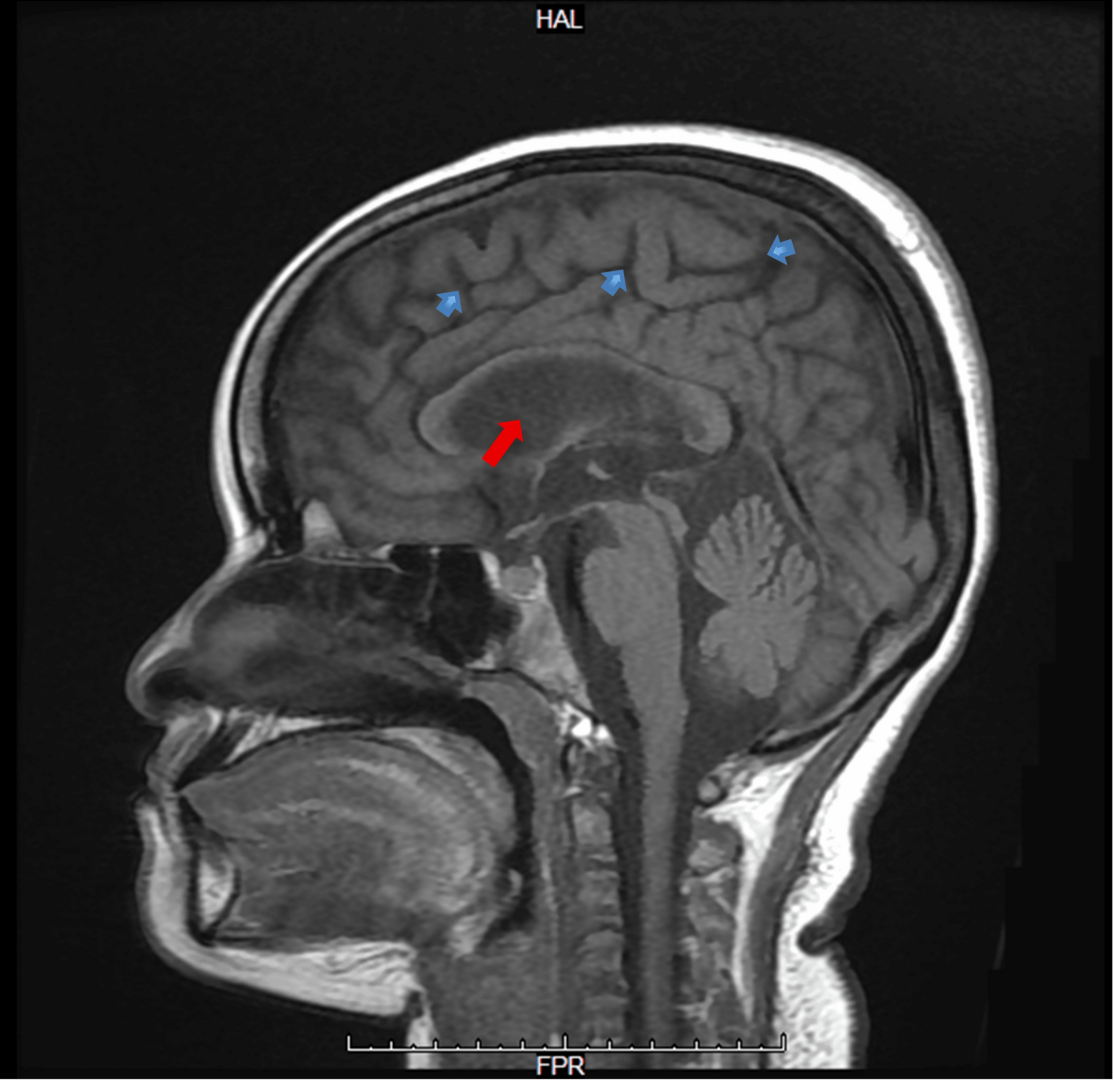
Sagittal T1-weighted MRI of the brain Sagittal T1-weighted MRI demonstrates the midline brain structures, including the corpus callosum, brainstem, and cerebellum. Cortical sulci appear widened (blue arrows), consistent with diffuse cortical atrophy. The ventricular system appears prominent for age (red arrow), in agreement with reported atrophic changes. The cerebellum and brainstem structures are preserved, with no evidence of mass effect or midline shift MRI: magnetic resonance imaging

Figures 1-3 show the findings of the first MRI brain without contrast, which reveals diffuse periventricular white matter high signal in FLAIR and T2 and low signal in T1 with no restriction in DWI. Other abnormal signal foci were seen in the bilateral thalami and peripheral pons, high signals in FLAIR and T2, low in T1, with no restriction in DWI.

Diffuse cortical atrophic changes were associated with a prominent ventricular system for the patient's age. The cerebellar hemispheres and vermis appeared normal, and there was no evidence of recent infarction, acute hemorrhage, or space-occupying lesion in the brain parenchyma, and no midline shift or mass effect. The pituitary gland and stalk appeared normal. The optic chiasm and optic nerves appeared normal. The cerebello-pontine angles also appeared normal. MRA showed no aneurysmal dilatation or significant narrowing.

**Figure 4:**
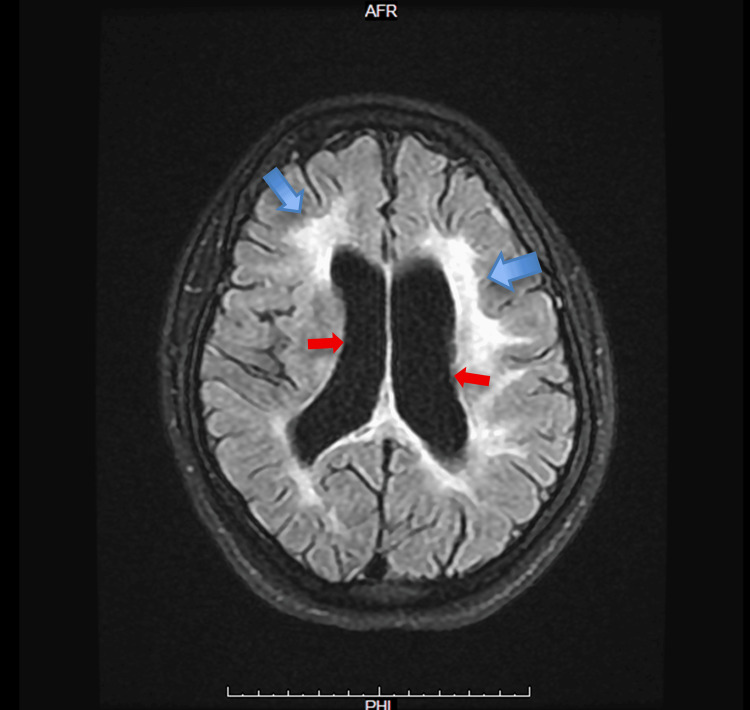
Axial T2 FLAIR MRI of the brain with contrast The axial T2 FLAIR sequence post-contrast demonstrates diffuse periventricular and deep white matter hyperintensities surrounding the lateral ventricles (blue arrows). The ventricles are enlarged relative to patient age (red arrows), consistent with reported cortical atrophy. No abnormal contrast enhancement is observed, suggesting the lesions are non-acute and non-enhancing. Findings are compatible with a demyelinating or inflammatory white matter process, as described in the clinical report MRI: magnetic resonance imaging; FLAIR: fluid attenuated inversion recovery

**Figure 5:**
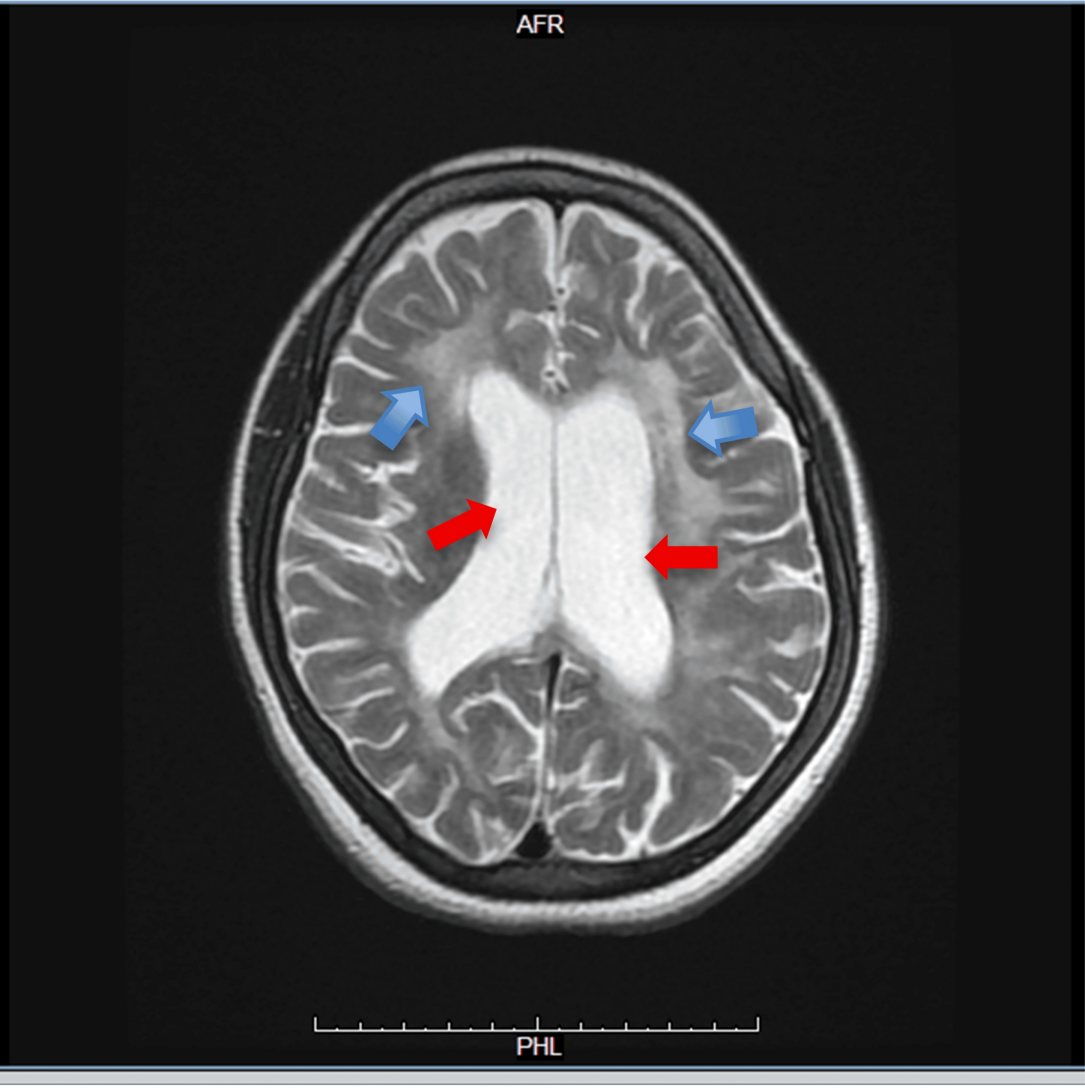
Axial T2 FSE-weighted MRI of the brain Axial T2-weighted MRI shows bilateral periventricular and deep white matter patchy hyperintensities (blue arrows) with enlarged ventricles (red arrows) and diffuse brain volume loss disproportionate to age. No restricted diffusion, hemorrhage, hydrocephalus, or mass effect is present. The findings are stable compared to previous MRI studies and are most consistent with chronic white matter involvement in neuropsychiatric SLE MRI: magnetic resonance imaging; FSE: fast spin echo; SLE: systemic lupus erythematosus

**Figure 6:**
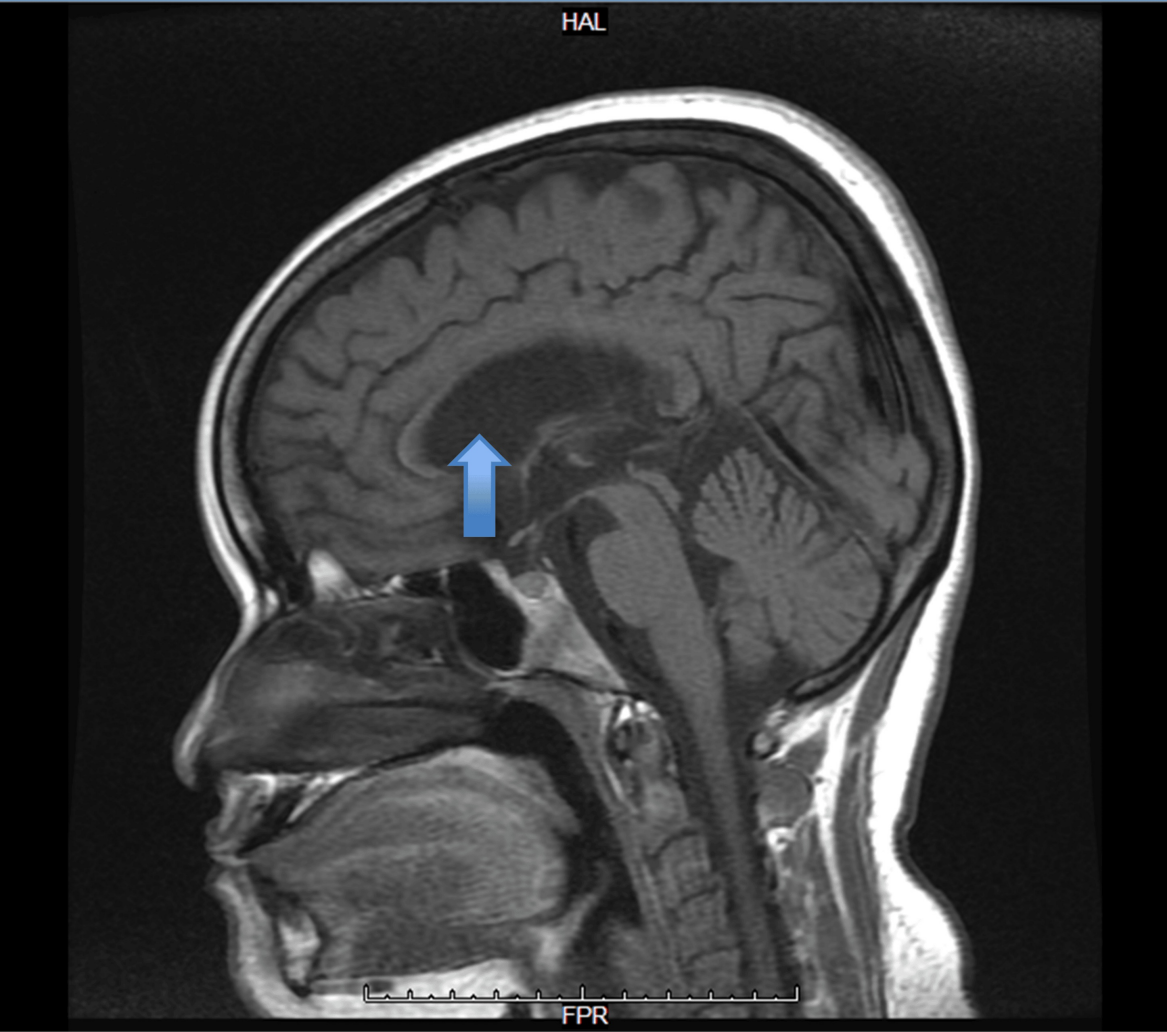
Sagittal T1-weighted MRI of the brain Sagittal T1-weighted MRI demonstrates diffuse cortical atrophy with enlarged ventricles (blue arrow) disproportionate to age. The midline structures, including the corpus callosum, brainstem, and cerebellum, are preserved. No midline shift, mass effect, or abnormal enhancement is present MRI: magnetic resonance imaging

An enhanced brain MRI with MRA performed in June 2025, when compared with the prior study from May 23, 2024, demonstrated stable bilateral periventricular and deep-white-matter FLAIR hyperintensities with diffuse brain volume loss (Figures 4-6); no new lesions, abnormal enhancement, mass effect, or vascular abnormalities were identified.

A multidisciplinary team - including neurology, psychiatry, and rheumatology - reached a consensus on the diagnosis of NPSLE, based on the combination of seizures, chronic psychiatric symptoms, elevated inflammatory markers, positive autoimmune serologies, and characteristic MRI findings.

The patient was initially treated with intravenous pulse corticosteroids, antiepileptics (levetiracetam), and hydroxychloroquine. Once stabilized, she received rituximab at a dose of 1 g intravenously. Following this treatment regimen, the patient exhibited significant clinical improvement, with cessation of seizures and better functional interaction. A follow-up contrast-enhanced MRI performed in June 2025 showed no interval progression of white matter lesions or cortical atrophy, confirming radiological stability (Figures 4-6). The patient remained clinically stable on a tapering dose of prednisone, hydroxychloroquine, and planned periodic rituximab therapy, with initial close monitoring every one to three months to assess neurological status and treatment response.

## Discussion

Literature review

Methodology

Search strategy: We also engaged in a structured and systematic approach to identify relevant literature concerning NPSLE, seizures, and brain atrophy. The search focused on studies published between 2013 and 2025 in peer-reviewed journals. Widely recognized scholarly databases were utilized, including PubMed, Google Scholar, Web of Science, and SpringerLink. To ensure consistency and replicability, the Preferred Reporting Items for Systematic Reviews and Meta-Analyses (PRISMA) guidelines were followed [11]. A comprehensive Boolean search string was applied:

("systemic lupus erythematosus" OR SLE) AND ("brain atrophy" OR "cortical atrophy" OR "hippocampal atrophy") AND (seizures OR epilepsy OR "neuropsychiatric lupus" OR NPSLE) AND (MRI OR "magnetic resonance imaging") AND ("case report" OR "case series" OR review)

This query yielded a total of 1,770 results. The authors screened titles, abstracts, and full texts when necessary to ensure relevance and methodological quality. Ultimately, 20 studies were selected for in-depth analysis and inclusion in this review.

Selection criteria: The eligibility of sources was assessed using predefined inclusion and exclusion criteria. These criteria were designed to align literature with the central research objective: investigating MRI-confirmed brain atrophy and seizure presentations in NPSLE. The inclusion criteria were as follows (1) studies published in English between January 2013 and January 2025, (2) peer-reviewed articles, (3) studies using an original research design such as case reports, case series, cohort studies, or systematic reviews, (4) studies focusing their examinations on MRI-confirmed brain changes and/or seizures in patients with SLE or NPSLE, and (5) studies available in full text either through institution subscription or open access. The exclusion criteria were as follows (1) papers written in a language other than English or outside the designated publication timeframe, (2) materials that are not peer-reviewed, such as editorials, commentaries, and opinion papers, (3) animal or in vitro research, (4) studies that do not include SLE patients or that have no CNS features, and (5) papers that could not be retrieved in full text or are behind a paywall.

**Figure 7:**
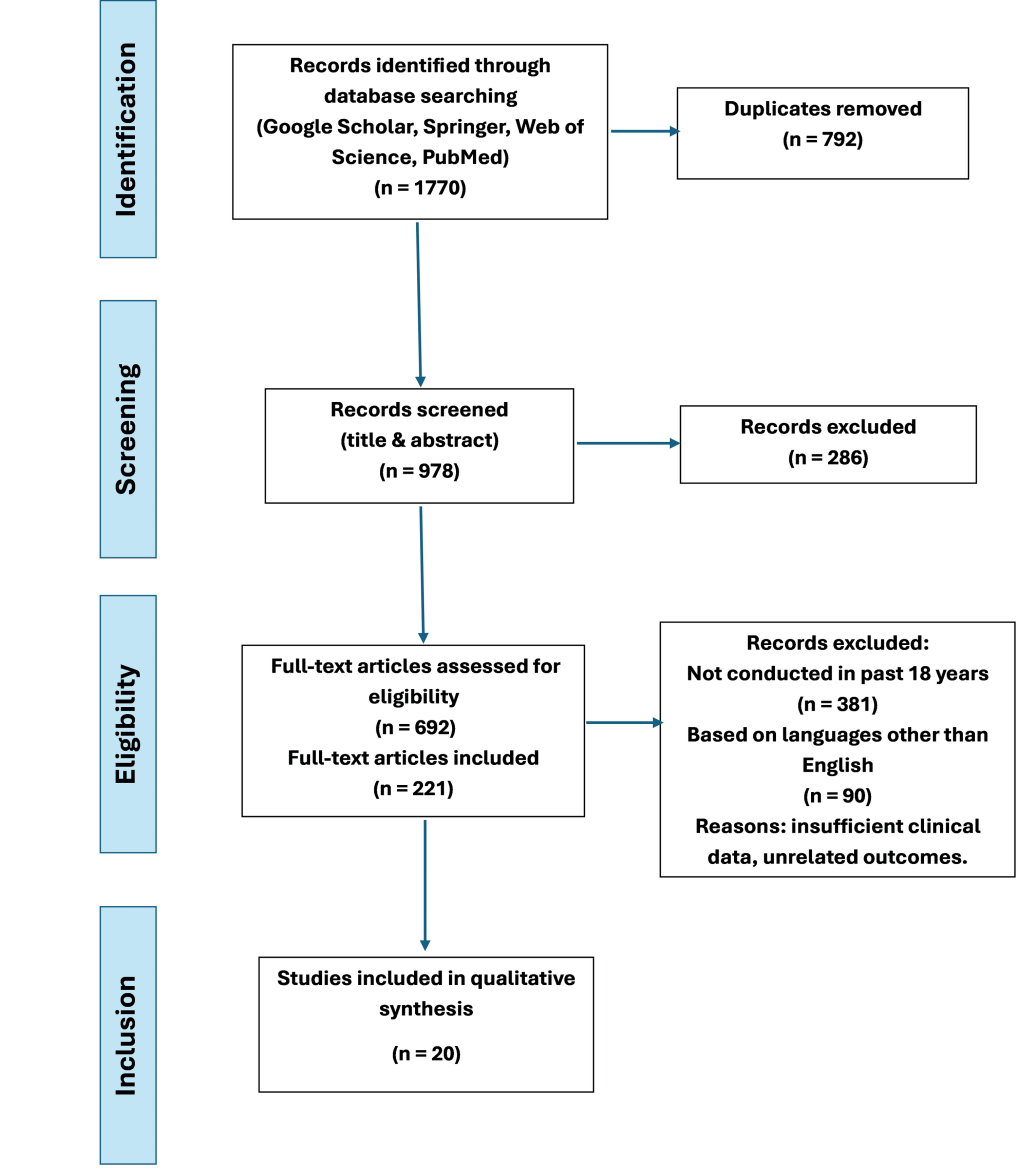
PRISMA flow chart depicting study selection PRISMA: Preferred Reporting Items for Systematic Reviews and Meta-Analyses

Data analysis: 20 articles were chosen based on the alignment of their titles and objectives with eligibility criteria and the inclusion of neuroimaging or seizure clinical data in SLE or NPSLE populations. A thematic analytic framework was adopted to distill and structure findings pertaining to structural brain alterations such as cortical or hippocampal atrophy, seizures in lupus, treatment strategies and their results, and MRI patterns that align with clinical severity. The studies’ findings were synthesized and juxtaposed with the clinical case of this study by using recurrent keywords and diagnostic criteria as categories. The systematic literature search and study selection process is further detailed in the PRISMA 2020 flow diagram, as shown in Figure 7.

Quality assessment: The methodological rigor and clinical relevance of each study were evaluated according to several quality indicators, including a clear definition of the patient population (SLE/NPSLE), the use of MRI or advanced neuroimaging techniques for diagnosis, and confirmation of seizure or other neuropsychiatric episodes. Additional considerations included adherence to PRISMA guidelines or other systematic methodologies, as well as the availability of full-text articles with complete clinical data. Priority was given to studies that provided longitudinal imaging findings, detailed treatment regimens, and clinical follow-up. This approach ensured that only high-quality, recent, and clinically applicable research was incorporated into the review, thereby strengthening the reliability of the discussion and the conclusions drawn. Also, the methodological quality was assessed using the Cochrane Risk of Bias Tool for randomized studies [12] and the Newcastle-Ottawa Scale (NOS) for observational studies [13], depending on study type.

As the findings of the risk-of-bias assessment reveal, the overall certainty of evidence across the 20 included studies was limited by study design. Most included articles were case reports or small case series, which are inherently prone to selection and reporting bias and do not allow adjustment for confounding. Among the observational cohort studies assessed with NOS, selection and outcome domains were generally adequate, while comparability was frequently “unclear” due to limited control for confounders. We did not identify any randomized trials pertinent to this review; therefore, the Cochrane Risk of Bias Tool did not apply to most included records. Taken together, the risk of bias across the evidence base is best characterized as moderate-to-high, and conclusions should be interpreted cautiously with emphasis on consistency of signals (seizure association, white-matter changes, and atrophy) rather than precise effect estimates.

Discussion and review

Neurological involvement in SLE is its most worrying manifestation; both neurological and psychiatric involvement can be of concern, due to the possible incipient damage. It ranges from cognitive disorders and simple mood changes to seizures and psychosis. In fact, seizures are very common and reported in 20% of patients, and they may even precede other systemic features of lupus, highlighting the potential for early neurological damage.

The pathway to NPSLE syndromes is multifactorial, including autoantibody-mediated neuronal injury, vasculopathy, upregulation of pro-inflammatory cytokines and downregulation of anti-inflammatory cytokines, and also BBB disruption [14]. Antiphospholipid antibodies, including anticardiolipin antibodies, have been associated with increased risk of thrombosis and seizures [9,15]. Our patient had positive anti-DNA antibodies and anticardiolipin antibodies and a negative infectious workup, supporting an autoimmune etiology. Especially with neuroimaging, MRI is an essential tool for diagnosing and monitoring NPSLE. Possible findings include hyperintense lesions in the periventricular and subcortical white matter, cerebral infarcts, cortical atrophy, and loss of hippocampal volume [8,16].

A meta-analysis confirmed that there were repetitive patterns of regional and global volume loss in both the frontal and parietal lobes among lupus patients [17]. Many studies show the correlation between these structural changes and cognitive impairment, even among individuals without significant neuropsychiatric symptoms [7,18]. Brain atrophy was a rare but significant finding in young SLE subjects who had long-standing or uncontrolled disease [2,5]. Hippocampal atrophy was also observed in both pediatric and adult SLE cohorts and correlated with memory deficits and poor academic performance [4]. The appearance of psychiatric symptoms, seizures, and imaging-confirmed diffuse cortex atrophy in our patient highlights the importance of being aware of this condition among the younger population. Longitudinal investigations suggest that some patients with NPSLE may either experience progressive atrophy of the brain or radiological stabilization depending on response to treatment [19,20].

Commonly utilized treatments for NPSLE may include corticosteroids, antimalarials, and immunosuppressants, with rituximab being promising in severe or refractory cases [1,21]. In our patient, MRI following treatment with rituximab showed radiological stabilization, consistent with previous reports that immunomodulation may slow or even stop progression of structural atrophy [10]. Seizures as a presenting symptom of NPSLE are well-described and may act as an early warning sign for CNS involvement. Rodriguez-Hernandez et al. note in their scoping review that seizures were most commonly associated with the diffuse subtype of NPSLE, and seizures tended to respond to corticosteroids and antiepileptics [3]. The literature also emphasizes that clinically prominent symptoms may still occur when imaging findings do not show gross abnormalities, possibly owing to subtle surface cortical atrophy and/or disturbed functional connectivity [2,6].

Lastly, neuroimaging follow-up becomes more relevant in pediatric and young adult populations, such as in the case of our patient. Unal et al. and Jeong et al. postulated that early radiological changes could precede the onset of cognitive symptoms or clinical seizures and thus activate treatment on time [22,23]. This accentuates the need for taking into account psychiatric history, autoimmune markers, and advanced neuroimaging techniques when encountering young patients afflicted with unresolved seizures or neurobehavioral disruptions. Our findings support the diagnostic-prognostic significance of brain atrophy and seizure onset in NPSLE. Hence, it fits the clinical course of our patients. Table 2 presents a comparative summary of major NPSLE-related literature that reports brain atrophy and seizure presentations, underscoring the spectrum of clinical manifestations, radiologic patterns, therapeutic regimens, and outcomes.

**Table 2:** Summary of selected case reports and reviews on brain atrophy and seizures in NPSLE NPSLE: neuropsychiatric systemic lupus erythematosus; MS: multiple sclerosis; MRI: magnetic resonance imaging; IVIG: intravenous immunoglobulin; CNS: central nervous system

Study	Year	Main clinical features	MRI findings	Treatment	Outcome/conclusion
Kalinowska-Lyszczarz et al. [2]	2018	Comparative study: MS vs. NPSLE	Distinct atrophy patterns: thalamus and basal ganglia in NPSLE	Not interventional	Demonstrated NPSLE has unique imaging biomarkers; aids differential diagnosis from MS and supports targeted imaging strategies
Julio et al. [9]	2025	Acute ventriculitis, status epilepticus	Inflammation of the ventricular lining, diffuse atrophy	High-dose steroids, immunosuppressants	Clinical stabilization achieved; highlights ventriculitis as a rare and severe form of NPSLE requiring aggressive therapy
Sarbu and Sarbu [10]	2020	Rapid neuropsychiatric deterioration	Fulminant brain atrophy, vasculitic vessel changes	High-dose corticosteroids, IVIG	Poor outcome with irreversible atrophy; the authors stress the value of vessel-wall imaging for identifying vasculitis NPSLE subtypes
Cheng and Hsu [14]	2025	Seizures, SLE-related psychosis, cognitive decline	Middle cerebral artery infarction, cortical atrophy	Corticosteroids, antipsychotics	Partial neurologic and psychiatric improvement; the authors emphasize early CNS recognition and immunotherapy to prevent irreversible damage
Mikdashi and Krumholz [15]	2023	Status epilepticus in SLE	No consistent imaging (meta-review)	Antiepileptics, corticosteroids	High variability: some patients recover fully, others develop chronic epilepsy; the authors call for EEG + MRI monitoring in acute seizures
Liu et al. [18]	2018	Asymptomatic or mild SLE without NPSLE	Global and hippocampal atrophy	Hydroxychloroquine, no immunosuppressants	Subclinical brain volume loss is common even in non-NPSLE; the authors propose brain atrophy as an early indicator of CNS involvement
Piga et al. [19]	2015	Progressive CNS involvement in SLE	Progressive cortical/subcortical atrophy over 20 years	Hydroxychloroquine-based long-term treatment	MRI shows CNS damage accrual despite stable peripheral disease; suggests disconnection between systemic and CNS markers
Kello et al. [20]	2019	Global cognitive decline in SLE	Suggestive of generalized volume loss (no MRI detail)	No active intervention (review focus)	Authors advocate for early cognitive trials in SLE due to the under-recognized burden; they suggest overlooked structural changes may underlie dysfunction
Syed et al. [21]	2024	Psychosis, seizure, mood instability	Cortical thinning, diffuse WMLs	Rituximab, corticosteroids	Rapid resolution of neuropsychiatric symptoms post-rituximab; the authors support its use in steroid-refractory NPSLE

NPSLE remains hard to diagnose and treat due to its heterogeneous manifestations, lack of a pathognomonic test, and variable neuroimaging findings. The case presented represents a rare but clinically significant phenotype of NPSLE in a young female, 22 years of age, who has symptoms of an early-onset psychiatric disorder that also includes seizures and the radiological features evidencing brain atrophy. While most patients with neuropsychiatric symptoms usually manifest those during or after the diagnosis of SLE, our patient had psychiatric manifestations for nearly a decade before the formal diagnosis. This atypical course highlights the need for early autoimmune screening among young people with unexplained behavioral and cognitive changes, particularly when laboratory findings such as ANA or anti-dsDNA positivity are suggestive.

Brain atrophy on the initial MRI was something unexpected for a young adult without prior neurodevelopmental disease or other risk factors for neurodegeneration. Atrophy is more often seen in elderly SLEs or those refractory to treatment [5,17]; however, cerebral volume loss has been proven early in new-onset cases [2,6]. Both hippocampal and cortical atrophy are correlated with neurocognitive deficits, which were evident in this patient's impaired memory loss, computation abilities, and social functions [4].

In this case, seizure activity necessitated hospitalization and prompted serial neuroimaging, which was crucial for both diagnosis and follow-up. Published literature reports a strong association between seizure activity and cortical or subcortical lesions, particularly in the presence of antiphospholipid antibodies [1,3]. In our patient, seizures were controlled with levetiracetam and corticosteroids, with no recurrence following rituximab infusion. This observation supports previous reports demonstrating the effectiveness of immunosuppressive therapy in seizure-related NPSLE [15,21]

An MRI profile, separated by a year, demonstrates radiological stability in the case. This is clinically relevant since a lot of longitudinal studies now tend to find that structural brain changes will come to their plateau or stabilize if, and only if, the inflammatory activity is controlled, especially with the likes of rituximab [10,19]. The unchanged ventriculomegaly and white matter hyperintensities are explained by the use of immunosuppressants at an early stage of the disease, which is in accordance with its relatively stable evolution. The psychiatric prodrome is an important piece of the puzzle, which was unrecognized for years. The literature shows that NPSLE can have psychiatric symptoms before it exhibits neurological or systemic features [14]. But in many of these cases, the psychiatric manifestation was erroneously taken as suggestive of a primary mental health disorder. This case highlights the necessity of interdisciplinary assessment and the use of neuroimaging and serology in young patients with treatment-resistant psychiatric symptoms.

## Conclusions

NPSLE is widely recognized for its diagnostic complexity and clinical heterogeneity. Our case, that of a young female with a decade-long history of neurobehavioral changes and recently seen generalized seizures, is an important reminder of the often subtle and insidious nature of neuropsychiatric disease recognition in SLE. The initial MRI finding of diffuse cortical atrophy is surprising in a woman of her age and also aligns with a chronic inflammatory process likely affecting the CNS. Our findings align with existing literature demonstrating that diagnosis relies on the integration of clinical assessment, serological markers, neuroimaging, and multidisciplinary collaboration. Early initiation of targeted immunotherapy, including rituximab, has been reported to stabilize both clinical symptoms and radiological progression, which we also observed in our patient. The literature we reviewed underscores that clinicians should maintain a high index of suspicion for NPSLE in young females presenting with seizures, psychosis, or unexplained cognitive dysfunction. Finally, our findings support published evidence indicating that severe neuroanatomical changes, such as cerebral atrophy, may stabilize with prompt and comprehensive therapy. Prospective studies are still needed to clarify the prognostic implications of brain atrophy and to optimize long-term treatment strategies for patients with NPSLE.
